# Incidence and characteristics of arterial thromboemboli in patients with COVID-19

**DOI:** 10.1186/s12959-021-00357-9

**Published:** 2021-12-20

**Authors:** Nancy Glober, Lauren Stewart, JangDong Seo, Christopher Kabrhel, Kristen Nordenholz, Carlos Camargo, Jeffrey Kline

**Affiliations:** 1 Indiana University, Indianapolis, Indiana United States; 2 Harvard Medical School, Boston, Massachusetts United States; 3 University of Colorado Anschutz Medical Campus, Colorado Aurora, United States; 4 Department of Emergency Medicine, Wayne State University School of Medicine, 4201 St. Antoine, 48201 Detroit, Michigan United States

## Abstract

**Background:**

Studies have reported COVID-19 as an independent risk factor for arterial thromboemboli.

**Methods:**

From a cross-sectional sample, we determined the incidence and location of arterial thromboemboli (myocardial infarction, ischemic stroke, peripheral artery), stratified by COVID-19 status, in the RECOVER database, which included data on patients at 45 United States medical centers in 22 states. Epidemiological factors, clinical characteristics and outcomes were collected through a combination of individual chart review and automatic electronic query and recorded in REDCap®. We investigated the association of baseline comorbidities on the development of arterial thromboemboli and analyzed results based on the presence or absence of concomitant COVID-19 infection, testing this association with Chi-squared. We also described use of anticoagulants and statins.

**Results:**

Data were collected on 26,974 patients, of which 13,803 (51.17%) tested positive for COVID-19. Incidence of arterial thromboemboli during hospitalization was 0.13% in patients who tested positive for COVID-19 and 0.19% in patients who tested negative. Arterial thromboemboli tended to be more common in extremities than in core organs (heart, kidney, lung, liver) in patients with COVID-19, odds ratio 2.04 (95% CI 0.707 – 5.85). Patients with COVID-19 were less likely to develop an arterial thrombus when on baseline statin medication (*p*=0.014). Presence of metabolic syndrome predicted presence of core arterial thrombus (*p*=0.001) and extremity arterial thrombus (*p*=0.010) in those with COVID-19. Arterial thromboemboli were less common in patients with COVID-19 than in those who tested negative for COVID-19.

**Conclusions:**

Presence of a composite metabolic syndrome profile may be associated with arterial clot formation in patients with COVID-19 infection.

## Introduction

Since the beginning of the coronavirus disease 2019 (COVID-19) pandemic, studies have reported increased incidence of arterial thromboemboli, prognosticating poor outcomes in patients infected with COVID-19.[1–5] Researchers speculated that SARS-CoV-2 infects endothelial cells through the angiotensin-converting enzyme 2 receptor leading to arterial thromboemboli.[6, 7] However, reports describing the incidence of arterial thromboemboli vary, [2, 7, 8], and the mechanisms leading to occurrence of arterial thromboemboli in those patients remain unclear.

Many studies investigating arterial thromboemboli are small retrospective studies or case series.[2, 9] As arterial thromboemboli are uncommon,[2] small studies are extremely limited in their ability to describe patterns in those patients. However, though the overall incidence is low, arterial thromboemboli remain a critical complication of infection with COVID-19. Thus, understanding factors contributing to arterial thromboemboli in patients with COVID-19 remains of great importance.

The purpose of this study was to query a large nationwide sample, the REgistry of suspected COVID-19 in EmeRgency care (RECOVER) database, in an effort to describe the incidence and location of arterial thromboemboli. We further described factors associated with the development of arterial thromboemboli and the effect of treatment with anticoagulants and statins. Specifically, we observed the incidence of comorbid metabolic syndrome as well as its associated risk for the development of arterial thromboemboli in COVID-19 infected patients.

## Methods

The RECOVER network collected data on Emergency Department (ED) patients at 45 United States medical centers who received diagnostic testing for SARS-CoV-2. Clinical factors and outcomes were documented for all patients with a positive swab molecular test or antibody testing within 30 days. A patient was considered positive for SARs-CoV-2 infection if either a molecular diagnostic test from a swab or serological IgM or IgG antibody test within 30 days was positive. A patient was considered negative for SARs-CoV-2 if in the absence of positive test results or clinical diagnosis of COVID-19 within 30 days. Patients were excluded if the test was done for automated or administrative purposes. Patients were only included in the database once, not multiple times in cases of multiple tests. The institutional review boards (IRBs) at all sites reviewed and approved the RECOVER registry protocol. A more detailed description of overall study methodology has been previously described.[10].

Each site collected data on at least 500 patients through a combination of automated electronic query and individual chart review by emergency medicine clinician-investigators using previously described methods.[10] Data were collected in REDCap®.

We recorded the incidence and location of arterial thromboemboli, stratified by COVID-19 status, and analyzed results using descriptive statistics. We investigated the association of various baseline comorbidities on the development of arterial thrombus and analyzed results based on the presence or absence of concomitant COVID-19 infection, testing this association with the Pearson’s Chi-squared. We also described patterns of use of different anticoagulants and statins. Analyses were done with SAS software 9.4 (SAS Institute, Cary, NC).

## Results

Data were collected on 26,974 patients, of which 13,803 (51.2%) tested positive for COVID-19. Of those included, 2,743 (10.2%) died, including 2,044 (74.5%) who tested positive and 699 (25.5%) who tested negative. We found significantly higher rates of positive COVID-19 testing among men (54.4% vs. 48.0%, *p*<0.001). Caucasian and African American patients were most commonly tested (Table 1). Patients who tested positive for COVID-19 were older and less likely to be of White race than those who tested negative (*P*<0.001, unpaired t-test and Chi-square, respectively). Of those with a positive COVID-19 test result, 265 (1.9%) were admitted to an observation unit, 2,581 (18.7%) were admitted to a regular or monitored floor, 4,278 (31.0%) were admitted to stepdown or progressive care, and 865 (6.3%) were admitted to an intensive care unit.

**Table 1:** Distribution of patient epidemiology and COVID-19 test results

	COVID-19 Test Results					
	**Negative**		**Positive**		**Total**	
Patient Characteristics	N (*n*=13,166)	% (48.8)	N (*n*=13,803)	% (51.2)	N (*n*=26,974)	% (100)
Gender						
Male	6098	45.6	7289	54.4	13,387	49.6
Female	7073	52.1	6514	48.0	13,587	51.2
Age (mean)	49.63 (SD 20.89)		56.51 (SD 19.46)			
Race						
Asian	392	1.5	414	1.5	806	3.0
Black	2923	10.9	4816	17.9	7739	28.7
Native Hawaiian or Other Pacific Islander	62	0.2	37	0.1	99	0.4
American Indian or Alaskan Native	84	0.3	62	0.2	146	0.5
Multiple Races	31	0.1	11	0.1	42	0.2
Unknown	1701	6.3	4504	16.7	6205	23.0
White	7959	29.5	3948	14.7	11,907	44.2

As noted in prior studies, we found an increased risk for new venous thromboembolism within 30 days in patients with severe COVID-19 infection, requiring hospitalization (*p*<0.001) (Table 2).

**Table 2:** Incidence of acute venous thromboemboli by hospitalization status in patients with or without COVID-19

COVID-19 Status	Venous Thromboemboli	Observation Admission *n*=1028 (%)	Regular or monitored floor admission *n*=9347 (%)	Stepdown or progressive care admission *n*=9018 (%)	Intensive care unit admission *n*=2623 (%)
Negative	**Yes**	7 (0.92)	168 (2.48)	79 (1.67)	85 (4.83)
	**No**	757 (99.08)	6599 (97.52)	4661 (98.33)	1674 (95.17)
Positive	**Yes**	3 (1.14)	66 (2.56)	64 (1.50)	45 (5.21)
	**No**	261 (98.86)	2514 (97.44)	4214 (98.50)	819 (94.79)

The incidence of arterial thromboemboli was 0.13% in patients who tested positive for COVID-19 and 0.19% in patients who tested negative. Patients with COVID-19 experienced a greater proportion of arterial thromboemboli in extremities (rather than core organs) as compared to those without COVID-19, although this did not reach statistical significance (Odds Ratio 2.04, 95% CI 0.71 – 5.85) **(**Table 3**)**. Two patients had two extremity arterial thrombi, whereas the others had one each. Only one patient had two core arterial thrombi, and the others had one each. All arterial clots were found in patients who were hospitalized within 30 days of COVID-19 testing.

**Table 3:** Incidence of arterial thromboemboli by type in patients with or without COVID-19

	COVID-19 Test Negative* n*= 38 (%)	COVID-19 Test Positive *n*= 25 (%)
**Arterial Extremity Thromboemboli**	**5**	**11**
Left arm	1 (2.6)	2 (8.0)
Left leg	1 (2.6)	3 (12.0)
Right arm	0 (0.0)	3 (12.0)
Right leg	3 (7.9)	3 (12.0)
**Arterial Core Thromboemboli**	**33**	**14**
Heart	13 (3.4)	6 (24.0)
Brain	19 (50.0)	6 (24.0)
Kidney	2 (5.3)	0 (0.0)
Intestine	0 (0.0)	2 (8.0)

Significantly more patients who tested positive for COVID-19 were started on some type of thromboprophylaxis. Of those who tested positive for COVID-19, 4,959 (36.1%) were started on thromboprophylaxis. Of those who tested negative for COVID-19, only 1,143 (8.7%%) were started on thromboprophylaxis (*p*<0.001). Aspirin use was documented in 4261 (15.9%) of patients. Arterial thromboemboli were documented in 25 (0.59%) of patients who were taking aspirin and 41 (0.22%) of patients not taking aspirin (*p*<0.001).

Disseminated Intravascular Coagulation (DIC) was diagnosed in a total of 29 (0.11%) patients, and incidence was not significantly different based on whether or not patients had a positive COVID-19 test (15 vs. 14, *p*=0.950). Ischemic stroke was diagnosed in 164 (0.61%) of all patients, 70 (0.53%) of whom had a negative COVID-19 test, and 94 (0.68%) of whom tested positive (*p*=0.115).

We investigated the association of various baseline comorbidities on the development of arterial thrombus and analyzed results based on the presence or absence of concomitant COVID-19 infection. We found a history of hypertension to be significantly associated with arterial thrombus, with patients with underlying hypertension having a significantly higher risk of developing core thrombi than patients without a history of hypertension, regardless of COVID-19 status (Chi-square, *p*=0.001 in COVID-19 positive and *p*=0.001 in COVID-19 negative). In COVID-19 positive patients, we also noted an association between hypertension and extremity thrombi (*p*=0.0174). The presence of baseline diagnosis of diabetes also was associated with arterial thrombi, although thrombus location depended on COVID-19 status. In COVID-19 positive patients, there was a significant association between diabetes and extremity thrombi (*p*=0.003), while COVID-19 negative patients were found to have an associated between diabetes and core thrombi (*p*=0.011). Hyperlipidemia was positively associated with both core and extremity thrombi, regardless of COVID-19 status. We found no association between obesity and arterial thrombi (Table 4).

**Table 4:** Association of comorbidities with arterial thrombi and COVID status

	Core arterial thrombus present		Extremity arterial thrombus present	
	**COVID-19 Test Positive**	**COVID-19 Test Negative**	**COVID-19 Test Positive**	**COVID-19 Test Negative**
**Hypertension**				
Yes	12	24	7	3
No	2	9	2	2
p-value	0.001	0.001	0.017	0.383
**Diabetes**				
Yes	6	13	6	1
No	8	20	3	4
p-value	0.100	0.011	0.003	0.944
**Hyperlipidemia**				
Yes	8	17	5	1
No	6	16	4	4
p-value	0.002	0.002	0.020	0.009
**Obesity**				
Yes	4	6	1	4
No	10	27	7	1
p-value	0.460	0.445	0.572	0.211

We created an additional composite parameter for metabolic syndrome, defined as patients with a documented diagnosis of at least three of the following four conditions: obesity (BMI >30 kg/m^2^), hypertension, hyperlipidemia, and diabetes, and examined the effect of metabolic syndrome in patients with arterial thromboemboli. Of the *n*= 61 patients with arterial thromboemboli, a total of 23 (37.70%) met criteria for composite metabolic syndrome diagnosis. Comparatively, 15.14% of the total population met criteria for metabolic syndrome.

The presence of metabolic syndrome was associated with the development of core arterial thrombus (*p*=0.001) and extremity arterial thrombus (*p*=0.010) in those with COVID-19. Conversely, the absence of metabolic syndrome was associated with core arterial thrombus in those without COVID-19 (*p*=0.001), but there was no difference in presence of extremity arterial thrombus in those without COVID-19 based on presence or absence of metabolic syndrome (*p*=0.330) (Table 5).

**Table 5:** Incidence of extremity or core arterial thromboemboli stratified by COVID-19 status and metabolic syndrome

	COVID-19 Test Negative N (%)	COVID-19 Test Positive N (%)
**Extremity Thromboemboli**	5	9*
With metabolic syndrome	0 (0.00)	4 (0.20)
Without metabolic syndrome	5 (0.05)	5 (0.04)
**Core Thromboemboli**	33	14
With metabolic syndrome	12 (0.57)	7 (0.35)
Without metabolic syndrome	21 (0.19)	7 (0.06)

*Two patients had incomplete history leading to inability to conclude whether or not the patients had metabolic syndrome

Finally, we examined the effect of statin therapy on arterial clots in those patients with metabolic syndrome. We excluded all patients for whom statin therapy status was undocumented (*n*=1,116, 4.15%). Of all patients included in the study, 5,880 (22.8%) were on statins and 19,830 (77.2%) were not. Of all patients with metabolic syndrome, *n*= 3,912 (71.14%) were on statins. Of all patients with metabolic syndrome who also had arterial thromboemboli, four were taking statins and one was not.

In patients who were COVID-19 positive, preexisting use of statins was associated with a lower incidence of arterial thromboemboli (*p*=0.014) (Table 6).

**Table 6:** Incidence of arterial clots in patients with and without COVID-19 as modified by use of statins

		Statin Use		Total
COVID-19 Status	Arterial Thrombus	Yes	No	
Negative	Yes	14 (0.24%)	21 (0.11%)	35
	No	3,042 (51.73%)	9,190 (46.34%)	12,232
	Sub total	3,056 (51.97%)	9,211 (46.45%)	12,267
Positive	Yes	9 (0.15%)	12 (0.06%)	21
	No	2,815 (47.87%)	10,607 (53.49%)	13,422
	Sub total	2,824 (48.03%)	10,619 (53.55%)	13,443
		5,880 (100%)	19,830 (100%)	25,710

## Discussion

This study represents the largest investigation to date of arterial thromboemboli in patients with COVID-19. As such, it adds to existing literature and better characterizes the extent of arterial thromboemboli complicating COVID-19 infection. Overall, we found that arterial thromboemboli were uncommon, precluding a more extensive analysis. However, we were able to describe common features of these patients. Interestingly, COVID-19 positive patients were more likely to sustain a limb arterial thrombosis than those who did not have COVID-19. While the mechanism behind this finding is unclear, this finding could inform understanding of comorbid conditions which predispose patients with SARS-CoV-2 to arterial thromboemboli.

In examination of patients’ baseline comorbidities, we found that three of the four components of metabolic syndrome (hypertension, diabetes and hyperlipidemia) likely mediated arterial clots, perhaps more significantly than COVID-19 infection status. This is a critical finding as metabolic syndrome is common [11] and is known to lead to an inflammatory and hypercoagulable state, causing endothelial dysfunction, and subsequent vascular remodeling and thrombosis.[11–13] We did not appreciate additive effects of metabolic syndrome and concurrent COVID-19 infection.

Interestingly, we did not find an association between obesity (the fourth component of metabolic syndrome) and arterial clots. Many studies have shown a paradoxical association of cardiovascular disease and obesity, in which obese patients with strokes or other cardiovascular disease have a better prognosis, including reduced mortality.[14–17] This paradox remains controversial and requires a higher quality of evidence to further clarify, but our results add to prior findings suggesting the need for distinction between obesity alone and a more complete metabolic syndrome profile.

Results of this large nationwide study indicate that baseline use of statins to control metabolic syndrome risk factors may lead to reduced risk of arterial thromboembolism in patients with new COVID-19 diagnosis, although overall numbers are small and need to be further studied.

## Limitations

This represents a retrospective secondary analysis of a prospective registry study. Not all patients were tested for arterial thromboemboli, as testing occurred at physician discretion. Therefore, it is possible that some arterial thromboemboli were not detected. Further, because the overall numbers of patients with arterial thromboemboli were so small, we were unable to perform a statistically meaningful multiple regression analysis, thus limiting our analysis to describing trends with Chi-squared tests. Potential bias exists in testing patients for COVID-19 because they required admission to the hospital. Patients admitted for ischemic stroke or myocardial infarction, for example, but with relatively few risk factors for COVID-19 infection could disproportionately fall into the COVID-19 negative cohort, inflating the incidence of arterial thromboemboli in that cohort.

## Conclusions

In a patient with newly diagnosed COVID-19 infection, there does not appear to be an increased risk of arterial thromboemboli. Presence of a composite metabolic syndrome profile may be associated with arterial clot formation.

## Data Availability

Data is available upon request.

## References

[1] Tang N, Li D, Wang X, Sun Z (2020). Abnormal coagulation parameters are associated with poor prognosis in patients with novel coronavirus pneumonia. J Thromb Haemost.

[2] Indes JE, Koleilat I, Hatch AN, Choinski K, Jones DB, Aldailami H, et al. Early experience with arterial thromboembolic complications in patents with COVID-19. J Vasc Surg. 2020. 10.1016/j.jvs.2020.07.089 PMC7452862 32861865

[3] Zhou F, Yu T, Du R, Fan G, Liu Y, Liu Z (2020). Clinical course and risk factors for mortality of adult inpatients with COVID-19 in Wuhan, China: a retrospective cohort study. Lancet.

[4] Guan WJ, Ni ZY, Hu Y, Liang WH, Ou CQ, He JX (2020). Clinical Characteristics of Coronavirus Disease 2019 in China. N Engl J Med.

[5] Huang C, Wang Y, Li X, Ren L, Zhao J, Hu Y (2020). Clinical features of patients infected with 2019 novel coronavirus in Wuhan, China. Lancet.

[6] Obi AT, Tignanelli CJ, Jacobs BN, Arya S, Park PK, Wakefield TW (2019). Empirical systemic anticoagulation is associated with decreased venous thromboembolism in critically ill influenza A H1N1 acute respiratory distress syndrome patients. J Vasc Surg Venous Lymphat Disord.

[7] Bellosta R, Luzzani L, Natalini G, Pegorer MA, Attisani L, Cossu LG (2020). Acute limb ischemia in patients with COVID-19 pneumonia. J Vasc Surg.

[8] Lameijer JRC, van Houte J, van Berckel MMG, Canta LR, Yo LSF, Nijziel MR (2020). Severe arterial thromboembolism in patients with Covid-19. J Crit Care.

[9] Abou-Ismail MY, Diamond A, Kapoor S, Arafah Y, Nayak L. Corrigendum to “The hypercoagulable state in COVID-19: Incidence, pathophysiology, and management” [Thromb. Res., 194, October 2020, Pages 101-115]. Thromb Res. 2020. 10.1016/j.thromres.2020.06.029 PMC7305763 32788101

[10] Kline J, Pettit K, Kabrhel C, Courtney D, Nordenholz K, Camargo C. Multicenter registry of United States emergency department patients tested for SARS-CoV-2. Journal of the American College of Emergency Physicians2020. 10.1002/emp2.12313 PMC7771823 33392542

[11] Franchini M, Targher G, Montagnana M, Lippi G (2008). The metabolic syndrome and the risk of arterial and venous thrombosis. Thromb Res.

[12] Dentali F, Squizzato A, Ageno W (2009). The metabolic syndrome as a risk factor for venous and arterial thrombosis. Semin Thromb Hemost.

[13] Prandoni P (2017). Venous and Arterial Thrombosis: Is There a Link?. Adv Exp Med Biol.

[14] Lavie CJ, Milani RV, Patel D, Artham SM, Ventura HO (2009). Disparate effects of obesity and left ventricular geometry on mortality in 8088 elderly patients with preserved systolic function. Postgrad Med.

[15] Lavie CJ, Milani RV, Ventura HO (2009). Obesity and cardiovascular disease: risk factor, paradox, and impact of weight loss. J Am Coll Cardiol.

[16] Forlivesi S, Cappellari M, Bonetti B (2021). Obesity paradox and stroke: a narrative review. Eat Weight Disord.

[17] Quiñones-Ossa GA, Lobo C, Garcia-Ballestas E, Florez WA, Moscote-Salazar LR, Agrawal A (2021). Obesity and Stroke: Does the Paradox Apply for Stroke?. Neurointervention.

